# Management of Osteoarthritic Axial Neck Pain With Cervical Neuromodulation

**DOI:** 10.7759/cureus.46890

**Published:** 2023-10-12

**Authors:** Naeem Haider, Akshat Gargya

**Affiliations:** 1 Anesthesiology, University of Vermont Health Network, Burlington, USA

**Keywords:** osteoarthritis, peripheral nerve stimulation, chronic pain, axial neck pain, neuromodulation

## Abstract

Chronic refractory facetogenic axial neck pain is a challenging diagnosis to manage long-term. With limited options available for patients who have failed cervical medial branch blocks, patients often have to endure a poor quality of life. Although neuromodulation devices such as peripheral nerve stimulators are currently available for the management of various chronic conditions, their role in the treatment of axial neck pain has not been studied. We present a case of successful management of facetogenic axial neck pain with cervical medial branch peripheral nerve stimulation.

## Introduction

Neck pain is a common presentation of cervical pathology in many patients. In any given year, it affects approximately 30% to 50% of adults, and in individuals with neck pain, 50% to 85% do not experience complete resolution of symptoms [1]. Axial neck pain due to osteoarthritis is characterized by dull pain located at or around the neck and upper back area [2]. Axial neck pain can arise from a variety of conditions, including muscles, joints, and ligaments [3]. Primary myofascial pain arises in the muscles themselves possibly due to a lower level of high-energy phosphates than in normal muscle tissue [3]. Secondary myofascial pain presents with degeneration of the facet joints or arthritis [3]. Pain is primarily aggravated by extension.

Several treatment modalities are currently available to manage neck pain from facetogenic sources ranging from physical therapy to medications, medial branch blocks, and radiofrequency ablation [4]. Axial neck pain can be difficult to control and can be very challenging to manage in refractory cases. In this case report, we present a patient who had failed medial branch blocks in the cervical spine and was implanted with a peripheral nerve stimulation (PNS) device, a neuromodulation modality to manage axial neck pain.

## Case presentation

A 60-year-old male patient with a past medical history of coronary artery disease, osteoarthritis, and emphysema presented to our pain clinic with bilateral neck pain for two years which started after a work-related neck injury where his face was pushed against a hard object. The pain was dull in character and rated 8/10 on the Numerical Rating Scale (NRS) even with minimal movement of the neck in either direction. The pain did not radiate and increased with rotating the head to the right and left, flexion, and extension of the neck. Physical therapy for six weeks and home exercise programs offered minimal improvement. He was using the muscle relaxant cyclobenzaprine three times daily for the last six months with minimal pain relief. In addition, he used acetaminophen and ibuprofen as needed along with a lidocaine patch (12 hours on and 12 hours off). These medications did not provide adequate pain relief. The patient had severe pain on facet loading on examination, and the Spurling test was negative. A recent magnetic resonance imaging had shown multilevel, high-grade, neuroforaminal narrowing due to uncovertebral hypertrophy and facet arthropathy from C3 to C7. The patient was scheduled for C4, C5, and C6 bilateral cervical medial branch block which provided pain relief but not enough to proceed with a second diagnostic and radiofrequency ablation. The patient experienced 50% pain relief in his symptoms which was below the threshold required by insurance companies to proceed with a second diagnostic medical branch block. In the clinic, the patient underwent a bilateral C4 cervical medial branch bilateral peripheral nerve stimulator with Sprint. At the two-month follow-up, the stimulating leads were removed, and the patient reported a significant decrease in his pain which was now 4/10. He reported an improved ability to move the neck and perform activities with minimal neck pain. The patient denied any complications from the procedure.

Technique

**Figure 1:**
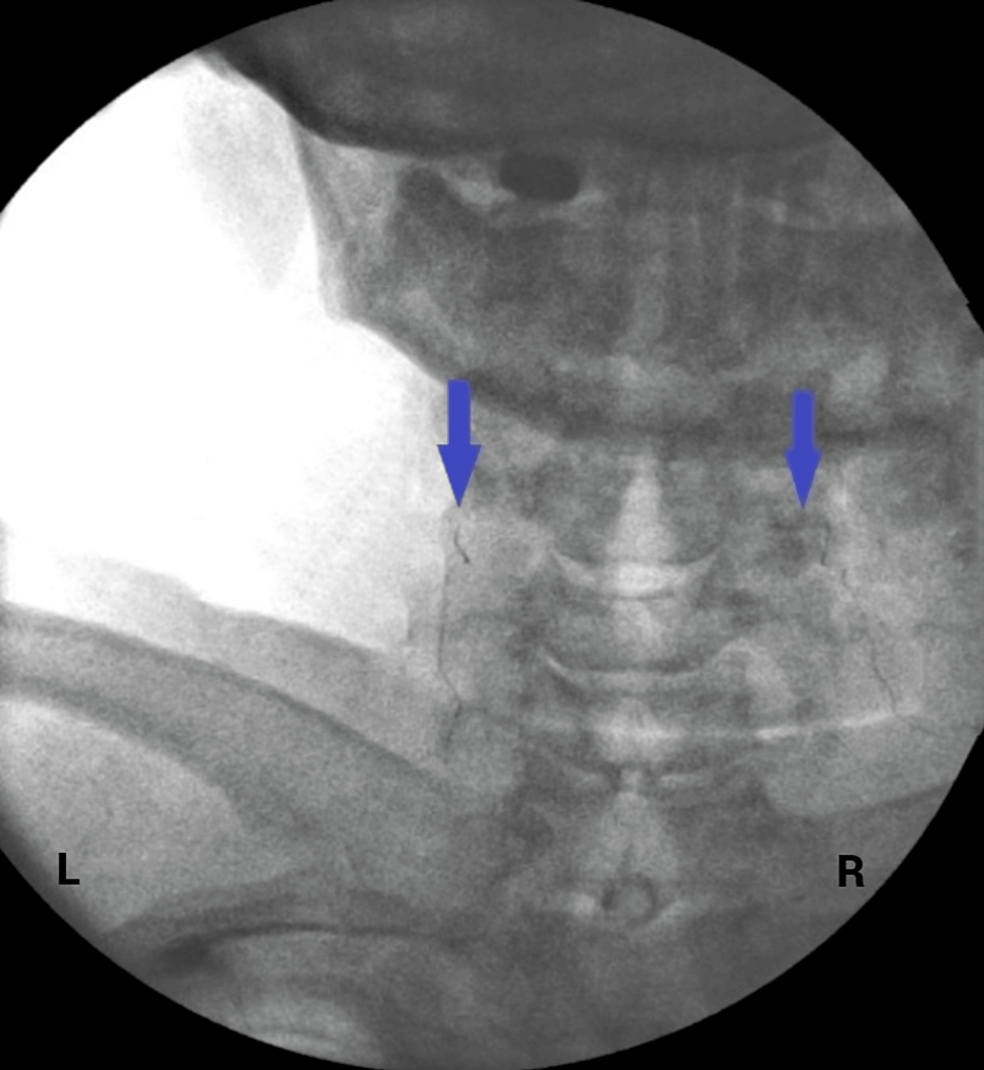
Anteroposterior fluoroscopic image of C4 vertebrae with final lead placement on both sides of the neck (blue arrow). L = left side of the neck; R = right side of the neck

**Figure 2:**
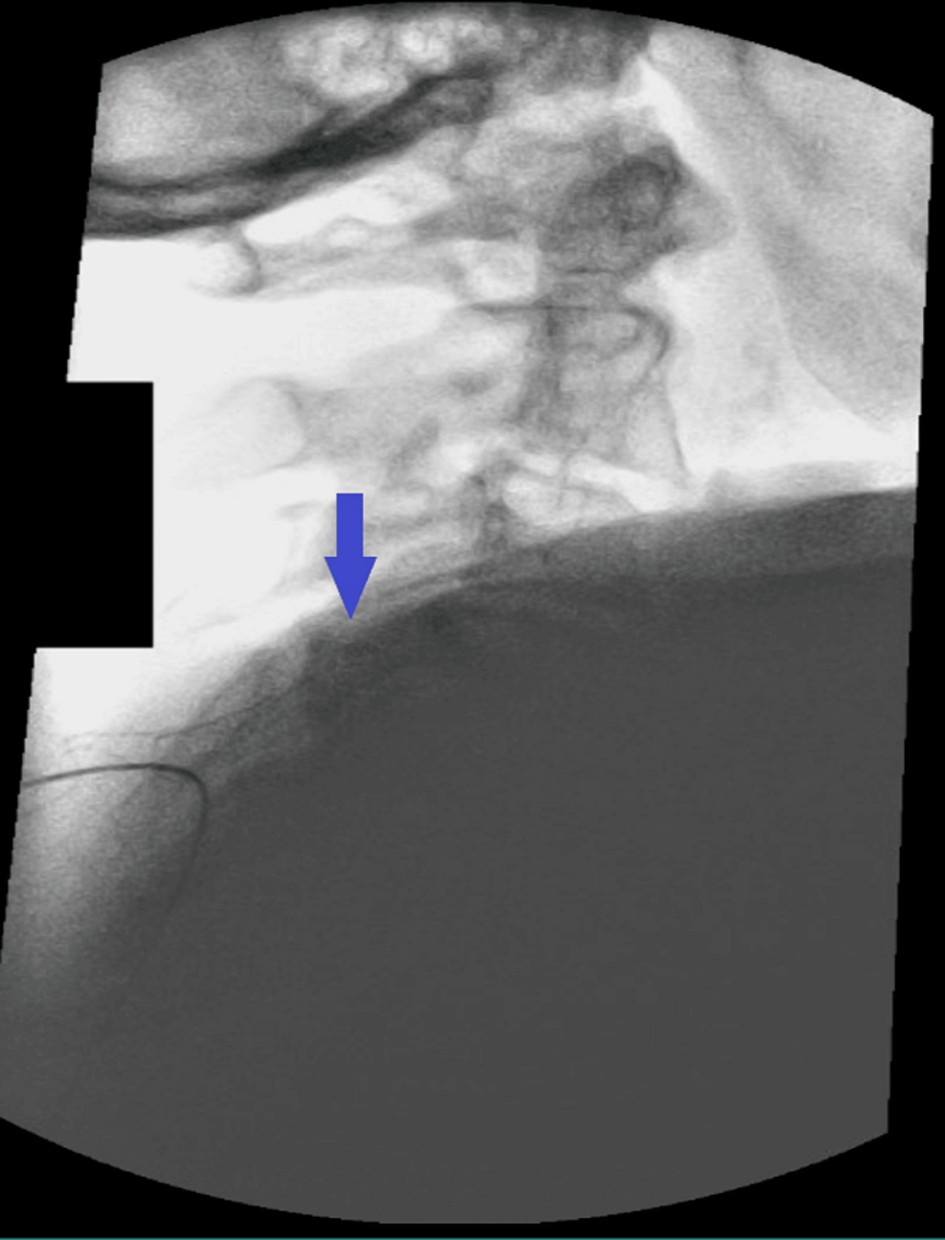
Lateral fluoroscopic image of C4 vertebrae with final lead placement (blue arrow).

The patient was positioned in a prone position on the procedure table and anatomical landmarks/body of C4 vertebrae were identified using fluoroscopy. Under sterile conditions, local anesthesia was injected over the skin and subcutaneous tissue using 1% lidocaine. A 17 G PNS stimulating needle with stylet was introduced in the anesthetized tract and was advanced until it reached the laminal surface approximately 6 cm deep to enable selective activation of large-diameter sensory fibers. A lateral view was then obtained to verify the needle position. At this point, stimulation was started to confirm a tingling sensation in the neck to cover the areas of the pain. Test stimulation was charge-balanced, asymmetric, and biphasic pulse train (100 Hz, 1-60 mA for 10-200 ms). Comfortable sensations were achieved at 60 mA. The stylet was then removed, and Sprint PNS fine wire coiled lead was inserted. The lead was positioned in place with minimal pressure and the introducer was removed. Final anteroposterior and lateral images were saved (Figures 1, 2).

Skin glue was applied to the skin to fix the lead and dressing was applied. An external, wearable pulse generator was then mounted on the upper back and attached to the lead. The patient reported no immediate complications and was able to tolerate the procedure.

## Discussion

Conservative treatments for axial neck pain include changes in lifestyle, cessation of smoking, and avoidance of neck-straining activities. Physical therapy is invaluable in the return of normal function. Interventional treatments may include medial branch blocks for pain originating in the facets followed by radiofrequency ablation [4]. Most cases of axial neck pain can be treated conservatively. Current medical treatment options for the management of osteoarthritic neck pain include conservative measures ranging from physical therapy to topical and oral anti-inflammatory medications [4]. If pain continues beyond six to eight weeks of conservative management, interventional procedures such as cervical medial branch blocks and subsequent radiofrequency ablation are utilized [4]. Often patients experience depreciating results from radiofrequency ablation (less than 50% pain relief). However, there are no guidelines for pain management in these patients and surgery may be the only treatment option [4]. In our case, we used PNS as a treatment option for our patient. We implanted the leads on the C4 level as this was with the mid portion of the patient’s pathology and correlated with the referral pattern of the pain related to his clinical history and examination.

PNS is currently being used for various conditions including radicular spinal and peripheral neuropathy, post-quadriceps tendon rupture, Complex regional pain syndrome, and chronic scrotal pain [5-10]. PNS has also been described to manage pain from osteoarthritis originating from facet joints in the lumbar area and knee [11,12]. Stimulation of the multifidus through a PNS device has been shown to be effective in providing pain relief in patients who have failed other treatments for low back/lumbar facetogenic pain, and data are currently available for long-term relief (up to 12 months) in these patient populations [13]. If our patient had reported insufficient pain relief after the removal of the leads at two months, we could have considered permanent PNS in the future.

PNS exerts its influence on both the peripheral and central nervous systems, including key cortical regions, and has demonstrated an ability to modulate inflammatory and autonomic pathways [14]. In a separate study, neuromodulation was found to enhance cortical activity in regions associated with pain and emotion. This was achieved by activating the dorsolateral prefrontal cortex on the same side as the stimulation and the sensorimotor cortex on the opposite side. Additionally, there were alterations in regional cerebral blood flow within areas of the brain related to central pain perception [15,16].

Patients with osteoarthritis frequently receive oral medications such as non-steroidal anti-inflammatory drugs and opioids to manage their condition. Unfortunately, these medications often come with undesirable side effects, including addiction, gastric ulcers, and kidney issues [17]. PNS offers a non-pharmacologic alternative to axial neck pain treatment. PNS currently possesses level II evidence supporting its use for facetogenic axial low back pain [18]. The application of PNS in the context of axial neck pain has not yet undergone evaluation [18]. Our case provides a novel treatment option for patients with refractory axial neck pain who are not candidates for radiofrequency ablation and surgery. To our knowledge, there are currently no reported cases in the literature of using PNS to manage osteoarthritis of the neck. Further research is needed to provide a more comprehensive understanding of its efficacy and safety profile, which can contribute to future advancements in the field of pain management.

## Conclusions

Cervical medial branch neuromodulation is a novel and promising approach for the treatment of chronic facetogenic axial neck pain. While conventional approaches such as physical therapy, medications, and medial branch blocks have been used, some patients continue to suffer from debilitating pain. The presented case demonstrates that cervical PNS may offer more than 50% pain relief and potential improvement in functionality in patients who have exhausted traditional treatments including failed cervical medial branch blocks. This research can be used as a starting point for further prospective trials on cervical medical branch neuromodulation, and future research to further delineate patient selection and possible benefits within the treatment algorithm for axial neck pain is required. As the field of pain management continues to evolve, innovative approaches such as PNS hold the promise of improving the quality of life for patients facing challenging and refractory conditions.
